# Phase-Transition Thermal Charging of a Channel-Shape Thermal Energy Storage Unit: Taguchi Optimization Approach and Copper Foam Inserts

**DOI:** 10.3390/molecules26051235

**Published:** 2021-02-25

**Authors:** Mohammad Ghalambaz, Seyed Abdollah Mansouri Mehryan, Ahmad Hajjar, Obai Younis, Mikhail A. Sheremet, Mohsen Saffari Pour, Christopher Hulme-Smith

**Affiliations:** 1 Metamaterials for Mechanical, Biomechanical and Multiphysical Applications Research Group, Ton Duc Thang University, Ho Chi Minh City 758307, Vietnam; 2 Faculty of Applied Sciences, Ton Duc Thang University, Ho Chi Minh City 758307, Vietnam; 3 Young Researchers and Elite Club, Yasooj Branch, Islamic Azad University, Yasooj 7591493686, Iran; alal171366244@gmail.com; 4 ECAM Lyon, LabECAM, Université de Lyon, 69005 Lyon, France; ahmad.hajjar@ecam.fr; 5 Department of Mechanical Engineering, College of Engineering at Wadi Addwaser, Prince Sattam Bin Abdulaziz University, Wadi Addwaser 11991, Saudi Arabia; oubeytaha@hotmail.com; 6 Department of Mechanical Engineering, Faculty of Engineering, University of Khartoum, Khartoum 11111, Sudan; 7 Laboratory on Convective Heat and Mass Transfer, Tomsk State University, 36 Lenin Avenue, 634050 Tomsk, Russia; sheremet@math.tsu.ru; 8 Department of Mechanical Engineering, Faculty of Engineering, Shahid Bahonar University of Kerman, Kerman 7616913439, Iran; mohsensp@kth.se; 9 Department of Materials Science and Engineering, KTH Royal Institute of Technology, SE-100 44 Stockholm, Sweden

**Keywords:** thermal energy storage, copper foam, phase transition, fast charging

## Abstract

Thermal energy storage is a technique that has the potential to contribute to future energy grids to reduce fluctuations in supply from renewable energy sources. The principle of energy storage is to drive an endothermic phase change when excess energy is available and to allow the phase change to reverse and release heat when energy demand exceeds supply. Unwanted charge leakage and low heat transfer rates can limit the effectiveness of the units, but both of these problems can be mitigated by incorporating a metal foam into the design of the storage unit. This study demonstrates the benefits of adding copper foam into a thermal energy storage unit based on capric acid enhanced by copper nanoparticles. The volume fraction of nanoparticles and the location and porosity of the foam were optimized using the Taguchi approach to minimize the charge leakage expected from simulations. Placing the foam layer at the bottom of the unit with the maximum possible height and minimum porosity led to the lowest charge time. The optimum concentration of nanoparticles was found to be 4 vol.%, while the maximu possible concentration was 6 vol.%. The use of an optimized design of the enclosure and the optimum fraction of nanoparticles led to a predicted charging time for the unit that was approximately 58% shorter than that of the worst design. A sensitivity analysis shows that the height of the foam layer and its porosity are the dominant variables, and the location of the porous layer and volume fraction of nanoparticles are of secondary importance. Therefore, a well-designed location and size of a metal foam layer could be used to improve the charging speed of thermal energy storage units significantly. In such designs, the porosity and the placement-location of the foam should be considered more strongly than other factors.

## 1. Introduction

The storage of heat energy in a material by inducing a phase change, and the resulting heat transfer mechanisms, are the subject of much current research and development [1]. This subject is of great interest in many engineering and industrial applications, such as buildings, solar collectors, power generation, and automotive applications [2,3,4]. Phase Change Materials (PCMs) can store/release a large amount of thermal energy when they undergo a phase change [5]. However, the rate of heat transfer in typical PCMs is limited by poor thermal conductivity [6]. Many researchers attempt to improve the heat transfer in PCMs by improving the geometrical design of the vessels in which the PCM is contained [7], adding fins [8] to increase the surface area available for heat transfer, adding nanoparticles to the PCM to produce nano-enhanced PCMs (NePCMs) [6,9], and embedding PCMs in thermal conductive metal foams [10]. 

Heat transfer to and from the environment during the phase change requires conjugate heat transfer in the enclosure walls and inside the PCM itself. Many PCMs undergo melting as their phase change in energy storage units: melting due to the influx of heat when excess energy is available, and solidifying to release heat when energy is extracted. Therefore, there is natural convection in molten regions of the PCM. A numerical study of conjugate heat transfer in a square enclosure filled with water that contained alumina nanoparticles, heated by a triangular region in one corner, found that the average Nusselt number in the system was directly proportional to the volumetric fraction of the nanoparticles and inversely proportional to the size of the triangular heat source [11]. This implies that convection becomes more important as the fraction of nanoparticles increases. The same authors have also explored the convective heat transfer of a similar fluid that was nonhomogeneous in a similar square enclosure with a central square region acting as a heat source/sink [12]. Their findings indicated that at low Rayleigh numbers, where laminar flow dominates, increasing the volume fraction of the nanoparticles and the size of the heating region directly resulted in increasing the Nusselt number and so enhanced the convective heat transfer, while the same changes gave adverse effects on convection at high Rayleigh numbers. A further study on a similar fluid and enclosure, with heat added in one corner and extracted at the opposite corner, was carried out [13]. There was a solid region in the center. The results showed that the ratio of thermal conductivities in the wall and the fluid and solid block size are the most significant factors for heat transfer optimization. This implies that both the geometry and nanoparticle fraction can be optimized to maximize the performance of thermal energy storage units. 

Zhou et al. [14] employed a machine learning approach and simplified the complicated energy storage process. Thus, a neural network could predict the energy storage behavior of the storage unit. Such a neural network model was used to design a hybrid energy system, including photovoltaic cooling and radiation cooling. Tang et al. [15] utilized the Taguchi method and optimized the thicknesses of the PCM layer to control nighttime cooling and daytime heat accumulation properly. The overall exergy was enhanced by 2.6% using the Taguchi method. The various novel optimization algorithms have been used to optimize the PCM-integrated systems is discussed in [16,17,18]. Moreover, the machine learning approaches have also contributed to modeling such systems [19,20] and have been reviewed in [21,22]. 

The introduction of fins in a cylindrical thermal energy storage system was shown to reduce the time to solidify the PCM by up to 30%, as well as reducing melting time [23]. Gürtürk & Kok [24] succeeded in reducing the melting time by 65% by adding fins designed using numerical modeling and validated with experiments. They found that if fins are too large, they impede convection and slow cooling. Corrugated fins have been shown to reduce solidification time in a cylindrical system, but only because the total surface area increases—the heat transfer per unit area is lower [25]. However, “triangular” fins (actually branches that have a triangular cross-section) running along the length of a cylindrical thermal energy storage unit reduce the solidification time by 38%, compared to a rectangular fin [7]. In a rectangular system, it has been shown that increasing the length of fins gives a higher heat transfer coefficient than increasing the number of fins [26]. Fins oriented horizontally disrupt convection, which is why vertical fins are much more common in the literature. Angled fins are also possible, and when two fins are used, the fastest heat transfer occurs when the fins were aligned at 45° to the horizontal. With three fins, the optimum angle is 60° [8]. Conversely, with Y-shaped fins, the angle and length of the branches and the overall length of the fin are inversely proportional to both the average temperature and the total energy of the system. When considering the shape of the fins and NePCMs, triangular fins with the long edge at the bottom of the thermal energy storage unit improve the melting rate [12].

Some studies have also analyzed the effects of combining fins and nanoparticles of different shapes. One such study found that for a system with fins that formed a “snowflake”, discharge time could be reduced by 15% by adding 4 vol.% platelet-shaped nanoparticles [14]. Another study showed that changing the length of the fin had the most significant effect when the nanoparticles were spherical, reducing melting time by 27% within the range of length studied, but that the fastest solidification occurred for platelet-shaped particles [27].

Introducing porous media in the enclosure of a thermal energy storage unit can considerably enhance the heat transfer rates. For example, making the walls of the enclosure porous and corrugated makes the heat transfer rate sensitive to the tilting of the enclosure [28]. The same study also showed that including two different types of nanoparticles increased the heat transfer rate, compared to a single type of nanoparticle. Increasing corrugation in a porous cavity also increases the strength of the NePCM [17]. A porous cavity also accelerates convection when heating is non-uniform [18]. However, if the nanoparticles are magnetic, an applied magnetic field can suppress convection [29]. Convection and heat transfer can also be increased by increasing the permeability of the PCM [30].

Incorporating metal foam into the PCM enhances the heat transfer. In one study, a metal foam increased the heat transfer by a factor of four at the expense of reducing the storage capacity by 13% due to the high value of pore density [31]. Wang et al. [32] experimentally studied the charging and discharging process of thermal storage systems containing copper foam and PCM. Mohammed et al. [33] investigated the melting heat transfer in a metal foam with heat generation. Since the heat was generated inside the porous structure, the melting time could be reduced by 21% compared to a localized heater. 

However, carefully designed fins have been shown to be as efficient as introducing metal foam in the system [34]. Melting time is also shown to be proportional to the porosity of an added foam but inversely proportional to the fraction of the enclosure that is filled with foam [35,36]. 

The addition of metal foam stimulated the melting process and resulted in decreasing the surface temperature of 47% for a PCM that was encapsulated in microscopic particles [37] and 38% for NePCM [38]. The reduction of the temperature was inversely proportional to the porosity. A combination of NePCM and metal foam was able to reduce the melting time by 90% [29] and the solidification time by 96% [30].

One study compared the effects of nanoparticles and metal foam directly. The addition of nanoparticles contributed 1.2% to melting time; the addition of metal foam reduced the melting time by 41.2% [39]. The marginal effects of nanoparticles can also be attributed to a reduction in both the convective and conductive heat transfer within the PCM [40]. A study that compared the influence of the foam porosity, the nanoparticle volume fraction, the Hartmann number of the PCM, and its Rayleigh number found that porosity was the dominant parameter [33].

The literature review shows that in each of the geometric designs of a PCM enclosure, the presence of each of the metal foams and nanoparticles can notably improve the heat transfer and response time of a thermal system. Using each of the nanoparticles or a porous matrix reduces an enclosure’s energy storage capacity and suppresses convection but may boost conduction. The present study aims to analyze the potential advantages of optimizing the design of a thermal energy storage unit in the presence of both nanoparticles and metal foams for the first time. An optimum design will highlight the true advantage of utilizing metal foams and nanoparticles for heat transfer in thermal energy storage units.

## 2. Mathematical Model

### 2.1. Model Description

**Figure 1:**
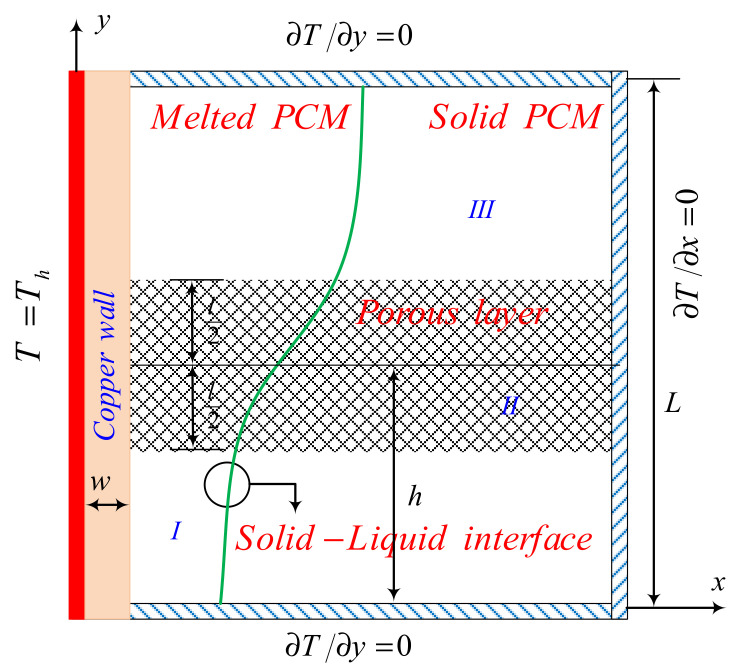
Schematic representation of the thermal energy storage unit.

A schematic demonstration of a latent heat thermal energy storage unit consisting of both clear and porous zones is provided in Figure 1. The porous zone comprises a copper foam with a porosity of ε filled by a nano-enhanced phase change material (NePCM), and the cavity size, *L* = 4 cm. 

There is a copper wall of thickness *w*, which is subject to a constant temperature of *T*_h_ and attached to the left side of the thermal energy storage unit. The other walls of the unit are well insulated, so they are assumed to permit zero heat flux. The thickness of the porous layer is *l*, and the distance between its center and the bottom wall is *h*. The local thermal equilibrium approach is used to model a copper foam filled by the NePCM composite. The matrix of the NePCM that is considered is capric acid, and the nanoparticles are copper. The properties of both materials are given in Table 1.

### 2.2. Physical Model and Governing Equations

The modeling assumptions are: (i) The variations of the density with the temperature can be modeled by the Boussinesq hypothesis, (ii) The NePCM and metal foam in the porous zone have the same temperature (local thermal equilibrium), and (iii) The NePCM and the solid matrix of the porous medium are isotropic and homogeneous. Considering these assumptions, the governing equations are:

(i) mass conservation

(1) $$\frac{\partial u_{j}}{\partial x_{j}}=0,$$

(ii) momentum conservation

(2) $$\varepsilon_{k}\frac{\partial u_{j}}{\partial t}+u_{i}\frac{\partial u_{j}}{\partial x_{i}}=-\frac{\varepsilon_{k}^{2}}{\rho_{\mathrm{NeP},l}}\frac{\partial p}{\partial x_{j}}+\frac{\varepsilon_{k}\mu_{\mathrm{NeP},l}}{\rho_{\mathrm{NeP},l}}\frac{\partial^{2}u_{j}}{\partial x_{i}\partial x_{i}}+\frac{\varepsilon_{k}^{2}}{\rho_{\mathrm{NeP},l}}f_{j},$$

where *t* is time and *x* and *y* are the positions. The dependent field variables are pressure, *p*, and the velocity vector, *u*. The porosity *ε*_k_ = *ε* inside the metal foam and 1 otherwise. The subscripts of “NeP,l” indicate the NePCM in the liquid state. The subscript *j* can be 1 for *x* or 2 for *y*; *k* is 1 for metal foam and 2 for clear flow. Both *j* and *k* are index parameters with no inherent physical meaning. The thermophysical properties are dynamic viscosity, *μ*, and density, *ρ*. Here *f* is a source term containing the body forces of the Darcy term, buoyancy forces, and a mushy zone control term. The porosity function and *f* are introduced as:

(3a) $$\varepsilon_{k}=\left\{\begin{array}{l} \varepsilon \;\mathrm{for}\;\mathrm{porous}\;\mathrm{layer}\\ 1\;\mathrm{for}\;\mathrm{clear}\;\mathrm{layers} \end{array}\right.,$$

(3b) $$f_{j}=-\frac{\mu_{\mathrm{NeP},l}}{K_{k}}u_{j}+\rho_{\mathrm{NeP},l}\beta_{\mathrm{NeP},l}\left(T-T_{m}\right)g_{j}-A_{\mathrm{mush}}\frac{{\left(1-\xi \left(T\right)\right)}^{2}}{\xi^{3}\left(T\right)+\varsigma },$$

*K_k_* is permeability, and it is artificially large outside the foam and equal to *K* inside the foam. *β* is the thermal expansion coefficient, and *g* is the acceleration due to gravity. *A*_mush_ is relatively large (~10^6^) compared to ϛ (~10^−3^). ξ(T) is the melt fraction. It is 1 for a fully liquid PCM and 0 when the PCM is fully solid. In the temperature range in which the PCM is expected to be partially melted, around the melting temperature, *T*_m_, ξ(*T*) is assumed to take a linear distribution. The permeability, melting fraction, and gravity acceleration are introduced by Equation (3c–e), respectively.

(3c) $$K_{k}=\left\{\begin{array}{l} K\;\mathrm{for}\;\mathrm{porous}\;\mathrm{layer}\\ \infty \;\mathrm{for}\;\mathrm{clear}\;\mathrm{layers} \end{array}\right.,$$

(3d) $$\xi \left(T\right)=\left\{\begin{array}{l} 0\;T<-\Delta T/2+T_{m}\\ \frac{T-T_{m}}{\Delta T}+\frac{1}{2}\;-\Delta T/2+T_{m}<T<\Delta T/2+T_{m}\\ 1\;T>\Delta T/2+T_{m} \end{array}\right.,$$

(3e) $$g_{j}=\left\{\begin{array}{l} 0\;j=1\\ g\;j=2 \end{array}\right.,$$

where Δ*T* is the temperature range over which the PCM is able to change phase. The permeability of the foam layer, *K*, is given in Equation (4a,b) as [43]:

(4a) $$K=d_{p}^{2}\frac{73\times 10^{-5}}{{\left(1-\varepsilon \right)}^{0.224}}{\left(1.18{\left(\frac{1-\varepsilon }{3\pi }\right)}^{0.5}\right)}^{-1.11}{\left[1-\exp\left(-\left(1-\varepsilon \right)/0.04\right)\right]}^{1.11},$$

(4b) $$d_{p}=254\times 10^{-4}\omega^{-1}\;\left(\mathrm{PPI}\right),$$

where *d*_p_ is the pore size, and *ω* is the pore density (pores per inch (PPI)). 

(iii) energy conservation for the NePCM domain:

(5) $$\frac{\partial T}{\partial t}+\frac{{\left(\rho C_{p}\right)}_{\mathrm{NeP},l}}{{\left(\rho C_{p}\right)}_{\mathrm{eff},k}}u_{j}\frac{\partial T}{\partial x_{j}}=\frac{\lambda_{\mathrm{eff},k}}{{\left(\rho C_{p}\right)}_{\mathrm{eff},k}}\frac{\partial^{2}T}{\partial x_{j}\partial x_{j}}-\frac{\rho_{\mathrm{NeP},l}h_{sf,\mathrm{NeP}}\varepsilon_{k}}{{\left(\rho C_{p}\right)}_{\mathrm{eff},k}}\frac{\partial \xi \left(T\right)}{\partial t},$$

where *C*_p_, *h*_sf_, and *λ* are the heat capacity, latent heat of phase change, and thermal conductivity, respectively. The subscript “eff” indicates the effective property for foam and the material inside the pores (composite property). The effective properties are introduced in Equations (6a,b) and (7), where the subscripts “sm” and “s” denote the copper foam and solid PCM, respectively.

(6a) $${\left(\rho C_{p}\right)}_{\mathrm{eff},k}=\left\{\begin{array}{l} \xi \left(T\right)\left(T\right){\left(\rho C_{p}\right)}_{\mathrm{eff},l}+\left(1-\xi \left(T\right)\left(T\right)\right){\left(\rho C_{p}\right)}_{\mathrm{eff},s}\;\mathrm{for}\;\mathrm{porous}\;\mathrm{layer}\\ \xi \left(T\right)\left(T\right){\left(\rho C_{p}\right)}_{\mathrm{NeP},l}+\left(1-\xi \left(T\right)\left(T\right)\right){\left(\rho C_{p}\right)}_{\mathrm{NeP},s}\;\mathrm{for}\;\mathrm{clear}\;\mathrm{layers} \end{array}\right.,$$

(6b) $${\left(\rho C_{p}\right)}_{\mathrm{eff},i}=(1-\varepsilon ){\left(\rho C_{p}\right)}_{\mathrm{sm}}+\varepsilon {\left(\rho C_{p}\right)}_{\mathrm{NeP},i},$$

(7) $$\lambda_{\mathrm{eff},k}=\left\{\begin{array}{l} \xi \left(T\right)\lambda_{\mathrm{eff},l}+\left(1-\xi \left(T\right)\right)\lambda_{\mathrm{eff},s}\;\mathrm{for}\;\mathrm{porous}\;\mathrm{layer}\\ \xi \left(T\right)\lambda_{\mathrm{NeP},l}+\left(1-\xi \left(T\right)\right)\lambda_{\mathrm{NeP},s}\;\mathrm{for}\;\mathrm{clear}\;\mathrm{layers} \end{array}\right.,$$

The effective thermal conductivity of the copper foam and PCM can be computed using theoretical models, as explained by Ranut [44]. Equation (8a,b) has been adopted to take into account the porous details [45,46].

(8a) $$\lambda_{\mathrm{eff},k}=\left\{\begin{array}{l} \xi \left(T\right)\lambda_{\mathrm{eff},l}+\left(1-\xi \left(T\right)\right)\lambda_{\mathrm{eff},s}\;\mathrm{for}\;\mathrm{porous}\;\mathrm{layer}\\ \xi \left(T\right)\lambda_{\mathrm{NeP},l}+\left(1-\xi \left(T\right)\right)\lambda_{\mathrm{NeP},s}\;\mathrm{for}\;\mathrm{clear}\;\mathrm{layers} \end{array}\right.,$$

(8b) $$\eta =\frac{1-\varepsilon }{3\pi },\;\Delta \lambda =\lambda_{\mathrm{sm}}-\lambda_{\mathrm{NeP},i},$$

Eenergy is conserved in the copper wall by adopting Equation (9).

(9) $${\left(\rho C_{p}\right)}_{w}\frac{\partial T}{\partial t}+=\lambda_{w}\frac{\partial^{2}T}{\partial x_{j}\partial x_{j}},$$

where subscript “w” denotes the wall.

The NePCM’s density is evaluated invoking a linear rule of mixtures (Equation (10a,b))

(10a) $$\rho_{\mathrm{NeP}}\left(T\right)=\rho_{\mathrm{PCM}}\left(T\right)+\upsilon_{\mathrm{na}}\left(\rho_{\mathrm{na}}-\rho_{\mathrm{PCM}}\right),$$

(10b) $$\rho_{PCM}\left(T\right)=\rho_{PCM,l}\xi \left(T\right)+\left(1-\xi \left(T\right)\right)\rho_{PCM,s},$$

in which *υ*_na_ is the volumetric concentration of nanoparticles in the PCM. The Brinkman model (Equation (10c)) is utilized to estimate the dynamic viscosity [47].

(10c) $$\mu_{\mathrm{NeP},l}=\mu_{\mathrm{PCM},l}{\left(1-\upsilon_{\mathrm{na}}\right)}^{-2.5},$$

The thermal expansion of the molten PCM is an important variable that drives the natural convection flow (Equation (11)):

(11) $$\rho_{\mathrm{NeP},l}\beta_{\mathrm{NeP},l}=\rho_{\mathrm{PCM},l}\beta_{\mathrm{PCM},l}+\upsilon_{\mathrm{na}}\left(\rho_{\mathrm{na}}\beta_{\mathrm{na}}-\rho_{\mathrm{PCM},l}\beta_{\mathrm{PCM},l}\right),$$

In accordance with previous literature [38], the Maxwell model (Equation (12)) was selected to compute the thermal conductivity of the NePCM.

(12) $$\frac{\lambda_{\mathrm{NeP},i}}{\lambda_{\mathrm{PCM},i}}=\frac{\lambda_{\mathrm{na}}\left(1+2\upsilon_{\mathrm{na}}\right)+2\lambda_{\mathrm{PCM},i}\left(1-\upsilon_{\mathrm{na}}\right)}{\lambda_{\mathrm{na}}\left(1+\upsilon_{\mathrm{na}}\right)+\lambda_{\mathrm{PCM},i}\left(2+\upsilon_{\mathrm{na}}\right)},$$

As with the density, the heat capacity of the NePCM is computed using a linear rule of mixtures, which is an implementation of the conservation energy (Equation (13a,b)).

(13a) $$\rho_{\mathrm{NeP}}C_{p,}{}_{\mathrm{NeP}}\left(T\right)=\rho_{\mathrm{PCM}}C_{p,}{}_{\mathrm{PCM}}\left(T\right)+\upsilon_{\mathrm{na}}\left(\rho_{\mathrm{na}}C_{p}{}_{,\mathrm{na}}-\rho_{\mathrm{PCM}}C_{p,}{}_{\mathrm{PCM}}\right),$$

(13b) $$\rho_{\mathrm{PCM}}C_{p,}{}_{\mathrm{PCM}}\left(T\right)=\rho_{\mathrm{PCM},l}C_{p}{}_{,\mathrm{PCM},l}\xi \left(T\right)+\left(1-\xi \left(T\right)\right)\rho_{\mathrm{PCM},s}C_{p}{}_{,\mathrm{PCM},s},$$

Lastly, the nanoparticles do not contribute to latent heat energy storage; thus, the latent heat of the NePCM is computed according to Equation (14).

(14) $$\frac{\rho_{\mathrm{NeP},l}h_{sf,\mathrm{NeP}}}{\rho_{\mathrm{PCM},l}h_{sf,\mathrm{PCM}}}=1-\upsilon_{\mathrm{na}},$$

The temperature continuity and heat balance were applied at the interface boundaries, which can be mathematically represented as Equation (15a,b).

(15a) $$\begin{array}{l} {\left.{\left[\xi \left(T\right)\lambda_{\mathrm{NeP},l}+\left(1-\xi \left(T\right)\right)\lambda_{\mathrm{NeP},s}\right]}_{I}\frac{\partial T}{\partial x_{j}}\right|}_{\;I}={\left.{\left[\xi \left(T\right)\lambda_{\mathrm{eff},l}+\left(1-\xi \left(T\right)\right)\lambda_{\mathrm{eff},s}\right]}_{II}\frac{\partial T}{\partial x_{j}}\right|}_{II}\\ {\left.T\right|}_{\;I}={\left.T\right|}_{II} \end{array}$$

(15b) $$\begin{array}{l} {\left.{\left[\xi \left(T\right)\lambda_{\mathrm{eff},l}+\left(1-\xi \left(T\right)\right)\lambda_{\mathrm{eff},s}\right]}_{II}\frac{\partial T}{\partial x_{j}}\right|}_{II}={\left.{\left[\xi \left(T\right)\lambda_{\mathrm{NeP},l}+\left(1-\xi \left(T\right)\right)\lambda_{\mathrm{NeP},s}\right]}_{III}\frac{\partial T}{\partial x_{j}}\right|}_{\;III}\\ {\left.T\right|}_{\;II}={\left.T\right|}_{III} \end{array}$$

where the domains *I*, *II*, and *III* were defined in the schematic Figure 1. Equation (15c–e) must be applied at the interface of the solid wall and the layers.

(15c) $$\begin{array}{l} {\left.\lambda_{w}\frac{\partial T}{\partial x_{j}}\right|}_{w}={\left.{\left[\xi \left(T\right)\lambda_{\mathrm{NeP},l}+\left(1-\xi \left(T\right)\right)\lambda_{\mathrm{NeP},s}\right]}_{I}\frac{\partial T}{\partial x_{j}}\right|}_{\;I}\\ {\left.T\right|}_{\;w}={\left.T\right|}_{I} \end{array}$$

(15d) $$\begin{array}{l} {\left.\lambda_{w}\frac{\partial T}{\partial x_{j}}\right|}_{w}={\left.{\left[\xi \left(T\right)\lambda_{\mathrm{eff},l}+\left(1-\xi \left(T\right)\right)\lambda_{\mathrm{eff},s}\right]}_{II}\frac{\partial T}{\partial x_{j}}\right|}_{II}\\ {\left.T\right|}_{\;w}={\left.T\right|}_{II} \end{array}$$

(15e) $$\begin{array}{l} {\left.\lambda_{w}\frac{\partial T}{\partial x_{j}}\right|}_{w}={\left.{\left[\xi \left(T\right)\lambda_{\mathrm{NeP},l}+\left(1-\xi \left(T\right)\right)\lambda_{\mathrm{NeP},s}\right]}_{I}\frac{\partial T}{\partial x_{j}}\right|}_{\;III}\\ {\left.T\right|}_{\;w}={\left.T\right|}_{III} \end{array}$$

Lastly, the boundary conditions at the outer walls of the unit can be formulated as Equation (15f,g).

(15f) $$\mathrm{At}\;\mathrm{the}\;\mathrm{adiabatic}\;\mathrm{wall}:\;u_{j}=0,\;\frac{\partial T}{\partial x_{j}}=0,$$

(15g) $$\mathrm{At}\;\mathrm{the}\;\mathrm{hot}\;\mathrm{wall}:\;u_{j}=0,\;T=T_{h},$$

### 2.3. Characteristic Parameters

The characteristic parameters of the present research are stored Energy in the unit (ES), the melt volume fraction (MVF), and charging power (CP). The total energy deposited in the foam-NePCM composite, including the latent and sensible energy, is the integration of energy change over the domain (Equation (16a)).

(16a) $$ES_{\mathrm{NeP}}=\int_{A,\mathrm{NeP}}{\left(\rho C_{p}\right)}_{\mathrm{eff},k}\left(T-T_{\mathrm{in}}\right)\;dA+\int_{A,\mathrm{NeP}}\rho_{\mathrm{NeP},l}h_{sf,\mathrm{NeP}}\varepsilon_{k}dA,$$

The sensible energy stored in the copper wall and the total energy stored in the unit are given by Equation (16b,c), respectively.

(16b) $$ES_{w}=\int_{A,w}{\left(\rho C_{p}\right)}_{w}\left(T-T_{\mathrm{in}}\right)\;dA,$$

(16c) $$ES_{t}=ES_{w}+ES_{\mathrm{NeP}},$$

Finally, the overall Melting Volume Fraction (*MVF*) is integrated over the PCM domain, taking into account the porosity of the porous layer (Equation (17)).

(17) $$MVF=\frac{\int_{A}^{}\varepsilon_{k}\xi \left(T\right)dA}{\int_{A}^{}\varepsilon dA},$$

In a practical application of a TES unit, the performance of the unit can be evaluated by the charging power (Equation (18)). This parameter shows the capacity of the PCM to store energy and depends on the amount of the energy stored at a 100% melting volume fraction.

(18) $$CP=\frac{ES\;\mathrm{when}\;\mathrm{the}\;\mathrm{melting}\;\mathrm{process}\;\mathrm{complete}}{\mathrm{full}\;\mathrm{melting}\;\mathrm{time}},$$

## 3. Solution Approach and Validation

### 3.1. Numerical Method

To investigate the melting performance of a partially porous square medium, the Galerkin finite element method (GFEM) is utilized. This method uses the weak form to solve the governing equations. Details of GFEM are described elsewhere [48,49]. The object variables, i.e., the velocity components in the *x* and *y* directions, pressure and temperature, are expanded by utilizing a shape function. The expanded equations were integrated over the simulation domain to form a set of residual equations. The second-order Gaussian-quadrature technique was used for numerical integration. An automatic time-step adjustment was applied in the form of the backward differentiation formula (BDF) [50] to ensure the convergence and accuracy of computations. The PARallel DIrect SOlver (PARDISO) solver [51,52,53] and the Newton-Raphson method with a damping parameter of 0.8 were utilized to reduce the output of the residual equations to ~10^−6^.

### 3.2. Impact of Mesh Size

The mesh quality can influence the accuracy of numerical solutions significantly. Thus, the impact of the mesh size of the solution was investigated systematically by adopting four different mesh sizes. The properties of the adopted meshes are presented in Table 2. The melt volume fraction, which shows the latent thermal energy storage, was selected as the characteristic parameter of the mesh study. The computations were performed for *L* = 40 mm, *w* = 3 mm, *l* = 6 mm, *h* = 11 mm, *ε* = 0.85, and *υ*_na_ = 0.06. 

**Figure 2:**
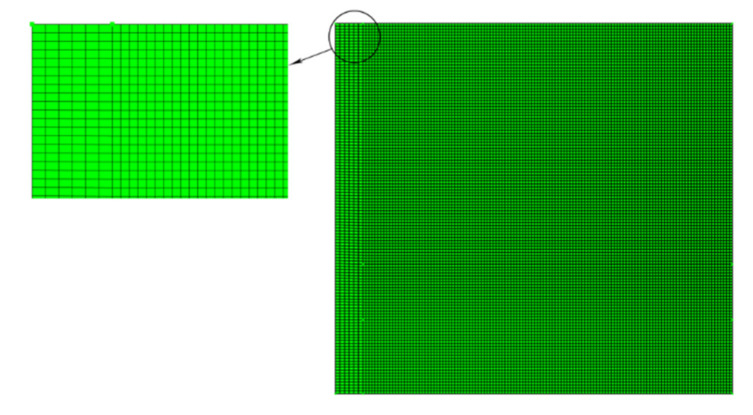
Schematic of the selected grid size 125 × 125 elements.

Figure 2 illustrates a view of the mesh used, which is a uniform structured mesh. The mesh in the PCM domain is slightly denser than in the solid wall: in the wall, there is only conduction heat transfer with smooth temperature gradients, and hence a dense mesh is not required for this domain. 

**Figure 3:**
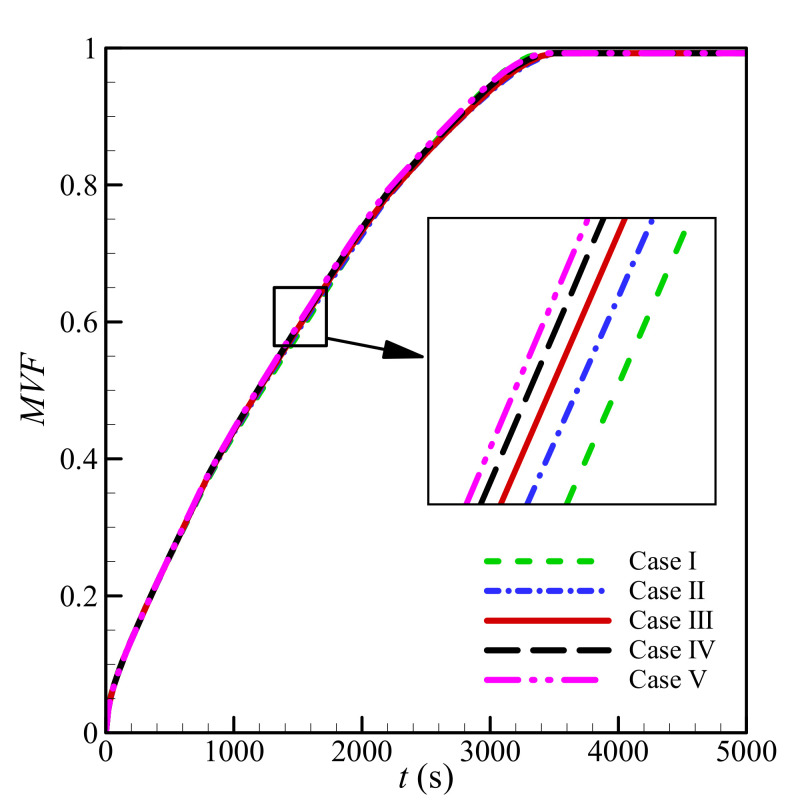
Dependences of *MVF* as a function of time on grid size when *L* = 40 mm, *w* = 3 mm*_,_ l* = 6 mm, *h* = 11 mm, *ε* = 0.85, and *υ*_na_ = 0.06.

Figure 3 depicts the time history of *MVF* for the selected mesh cases of Table 2. Figure 3 shows that the variation of the mesh changes the results slightly. The corresponding computational time is reported in Table 2. As seen, the computational time for Case I is higher than Cases II-IV. The reason is that a low-resolution mesh cannot adequately capture a narrow phase change interface, and thus the solver must select small time steps to keep the solution accurate and stable. This leads to a longer simulation, despite the coarser mesh. For Case II, the computational time drops, since the mesh-size only increased slightly, but could capture the behavior of the phase change region more conveniently, allowing larger time steps. Any further increase in mesh size increases the computational time. Since the change in *MVF* is minimal with the increase of mesh size, Case III shows a mesh size of 125 × 125 elements (PCM domain) and 6 × 125 elements (wall) for the computations to minimize the computational costs while maintaining an acceptable level of accuracy.

### 3.3. Validation

The results of the present model are validated through a comparison with the investigation of Al-Jethelah et al. [39] for the melting of coconut oil enhanced by copper oxide nanoparticles, embedded in an aluminum foam. The copper oxide-coconut oil system is a stable NePCM that could stay stable for months with good phase change cycling properties. The composite was embedded in a rectangular thermal energy storage unit 5.0 cm wide and 7.2 cm high. One edge was subject to a uniform heat-flux while the other surfaces were at zero heat flux. The experiment was performed for a foam porosity of 0.92. The phase change temperature of PCM was 24 °C, and the heating commenced when the NePCM composite was at an initial uniform temperature of 20 °C. The permeability of the foam was evaluated to be 3.3 × 10^−7^ m^2^ (Equation (4a,b)).

**Figure 4:**
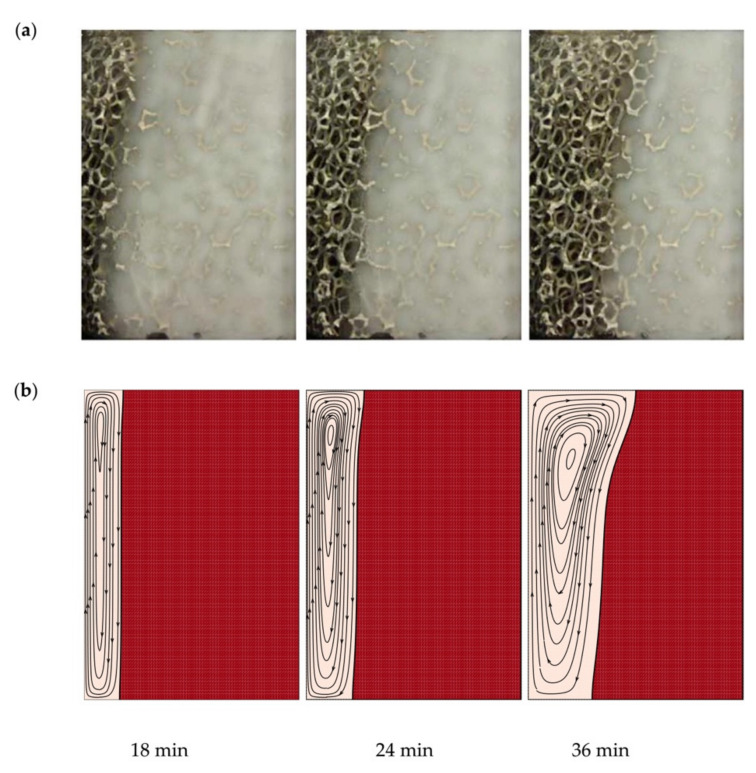
Comparison of the melting interface between the numerical results of the present study and published experimental results. (**a**) shows the images of the melting field at various time steps, reported in [39]. (**b**) shows the computed melting field of the present simulations. Adapted with permission from ref. [39]. Copyright 2019 Elsevier.

Figure 4a shows the images of the melting field at various time steps, reported in [39]. Figure 4b shows the computed melting field of the present simulations. Both images show the significance of natural convection flows in open foams. Moreover, the melting fronts for both images are the same, which implies that the model can reproduce the behavior of the system accurately.

**Figure 5:**
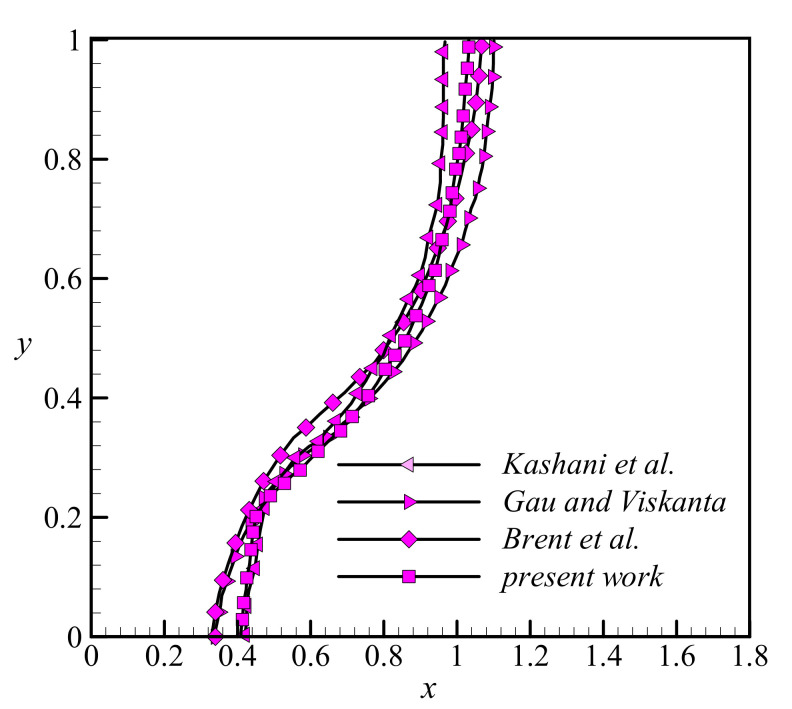
A comparison between the results of the present study and the published literature. Data from [54,55,56].

The melting front was also computed according to the conditions of benchmark references for a case with a Rayleigh number *Ra* = 1.25 × 10^5^ and a Prandtl number *Pr* = 50 [54,55]. This case shows the melting of a PCM in a rectangular cavity when the heated wall is subject to a uniform hot temperature. The melting fronts are plotted in Figure 5 for all cases, which denotes fair proximity between all captured melting fronts. The melting front computed by Kashani et al. [56] has also been added for the sake of comparison.

## 4. Results and Discussion

The key parameters are the thickness of the copper wall (0 ≤ *w* ≤ *3L*/25), the thickness of the porous layer (0 ≤ *l* ≤ *L*/5), the height of the porous layer (*L*/5 ≤ *h* ≤ 4*L*/5), the porosity of the porous layer (0.8 ≤ *ε* ≤ 1), and the nanoparticle’s volume concentration (0 ≤ *v*_na_ ≤ 0.08).

Taguchi’s technique is a robust statistical method used to optimize the design of experiments in which several parameters or control factors influence the outcome by performing the minimum number of required tests and reducing the associated experimental costs. For the present thermal energy storage unit, the five key parameters are considered to be *control factors*, and the value of each parameter, or *level*, can vary among different values. Here, each control factor is assigned four levels. The five control factors and their levels are presented in Table 3.

If all the possible combinations were to be tested, 4^5^ = 1024 experiments would be required, which is a prohibitive number. Taguchi’s method is used to reduce the number of required tests, and the L16 orthogonal array concept is utilized (Table 4). As can be seen in that table, only 16 tests with different combinations of the control factors are sufficient to define the influence of the factors, based on Taguchi’s algorithm. 

Once the required tests are defined, the next step is to perform an analysis of the signal to noise ratio, S/N ratio or SNR, in order to evaluate the effect of each parameter on the outcome by calculating the ratio of the signal (desired outcomes) to the noise (undesired outcomes). Three different optimization criteria can be adopted, depending on the result: the higher-the-better, the nominal-the-better, and the lower-the-better. In the present investigation, the time required for full melting, *t*|*_MVF_*_=1_, is the result that should be optimized. As the objective is to reduce *t*|*_MVF_*_=1_ to achieve more efficient thermal storage, then the *lower-the-better* approach is appropriate. In addition, a linear regression equation is derived to create a simple predictive relationship between *t*|*_MVF_*_=1_ and the various control factors:

*t*|*_MVF_*_=1_ (s) = −5100 − 10.0 *w* (*mm*) − 215.0 *L* (*mm*) + 177.50 *h* (*mm*) + 9600 *ε* − 11250 *υ*_na_, (19)

Table 4 and Equation (19) clearly indicate that all the control factors have a non-negligible influence on the result. Using Equation (19), the optimal control factors may be derived and are presented in Table 5. The optimized factors are: *w* = 4 mm, *l* = 8 mm, *h* = 11 mm, *ε* = 0.8, and *υ*_na_ = 0.04, which gives melting time, *t*|*_MVF_*_=1_ = 2025 s, while performing a test with optimal condition that gives *t*|*_MVF_*_=1_ = 3291.6 s. This value of *t*|*_MVF_*_=1_ is lower than that of all the 16 combinations performed earlier, indicating that the factors are likely to be optimized. The optimum case shows a 58% reduction in melting time, 100 × (7900−3291.6)/7900, compared to the design case of no. 9 of Table 4. 

**Figure 6:**
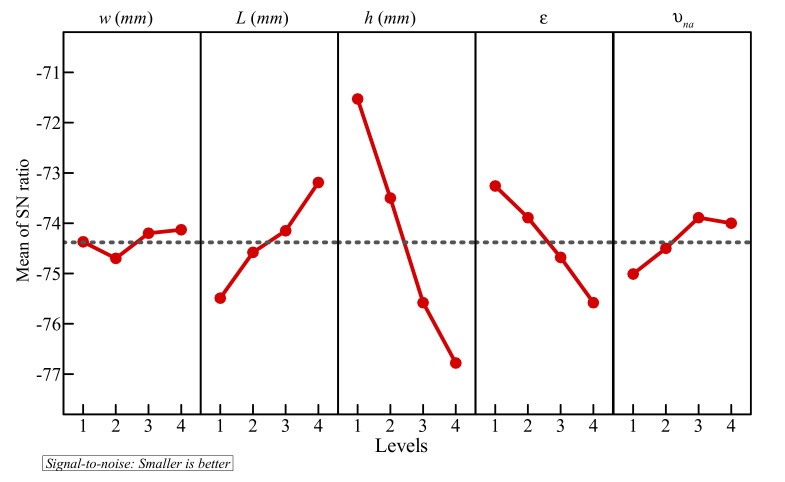
Signal to noise ratios of tested levels: the optimum levels are: *w* = 4, *L* = 4, *h* = 1, *ε* = 1, and *υ*_na_ = 3.

For now, it was observed that all the factors influence the result. However, the degree of influence of all the parameters is not necessarily the same. This can be evaluated by analyzing the SNR associated with each control factor. Table 6 presents the mean values of SNR for the different levels of each factor. The variation of SNR with the level of the factors is also illustrated in Figure 6, which shows that the optimal control factors that were previously obtained provide the highest values of SNR. In Table 6, the difference between the maximum and minimum values of SNR is calculated and denoted δ, which indicates the importance of each factor. As a consequence, the control factors can be ranked from the most influential to the least influential. The influence of the factors on the results has, therefore, the following decreasing order: *h* > *ε* > *l* > *υ*_na_ > *w*. This indicates that the properties of the porous layer, i.e., its height, porosity, and thickness, are the most significant parameters, followed by the volume fraction of the dispersed nanoparticles, while the thickness of the copper wall is the least significant parameter.

Twelve more experiments were performed to investigate the optimum control factors further (Table 7). The volume fraction of the nanoparticles was constant at 0.04. From the remaining control factors, three were constant, while the fourth was varied to three different values in each group of experiments. The optimal time for complete melting from the previous tests, *t*|_optimum_ = 3291.6 s, was taken as the test time, and the melted volume fraction was measured at this time for each experiment. A volume fraction of melt that is lower than 1 indicates that full melting is not yet achieved, and the time required for full melting in the experiment is greater than *t*|_optimum_. It can be seen in Table 7 that in 10 out of 12 experiments, the PCM has not fully melted at *t*|_optimum_, which supports the supposition that the parameters that were calculated previously are indeed optimized. In the remaining two experiments, complete melting is fully achieved at *t*|_optimum_, when *w* was increased from 4 mm to 6 mm and when ε was increased from 0.8 to 0.85. Furthermore, the difference between the obtained values for melting time and *t*|_optimum_ is lower when *w* is varied, which supports the rank defined based on Table 6. The variation of the foam layer’s location from 2 mm to 6 mm increased the MVF from 0.917 to 0.998, which shows an 8.8% improvement in the melting rate. 

In order to provide a physical explanation for the results, various graphical representations of the streamlines and isotherms in the cavity, as well as the time evolution of the melted volume fraction *MVF* and of the stored energy *ES*, are presented.

**Figure 7:**
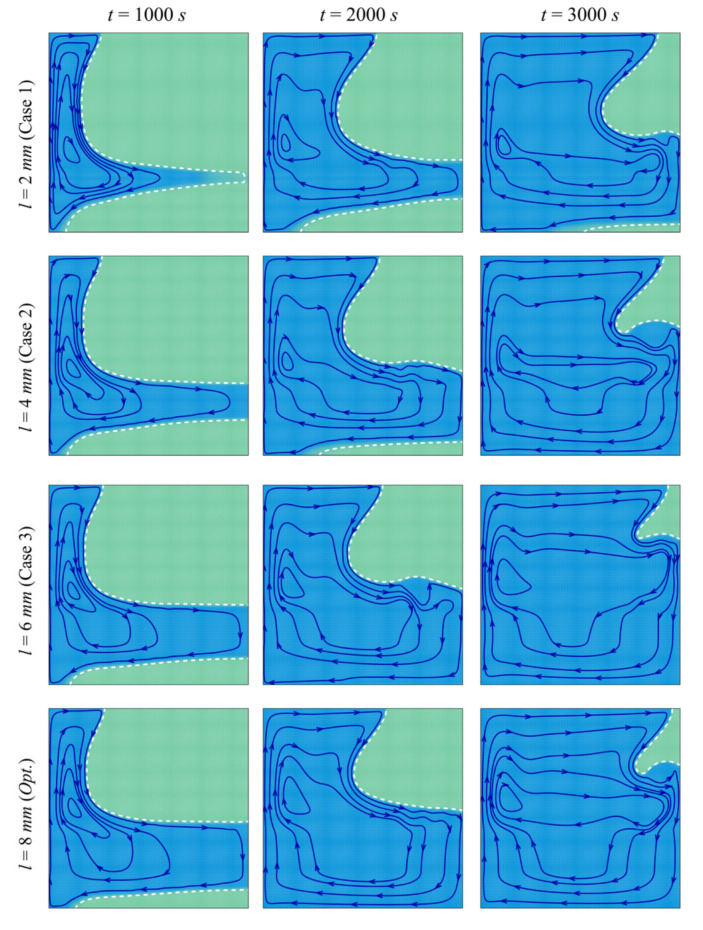
Streamlines and interface of melting (white dashed line) for different control parameters at three times when *L* = 40 mm, *w* = 4 mm*_,_ h* = 11 mm, *ε* = 0.80, and *υ*_na_ = 0.04.

**Figure 8:**
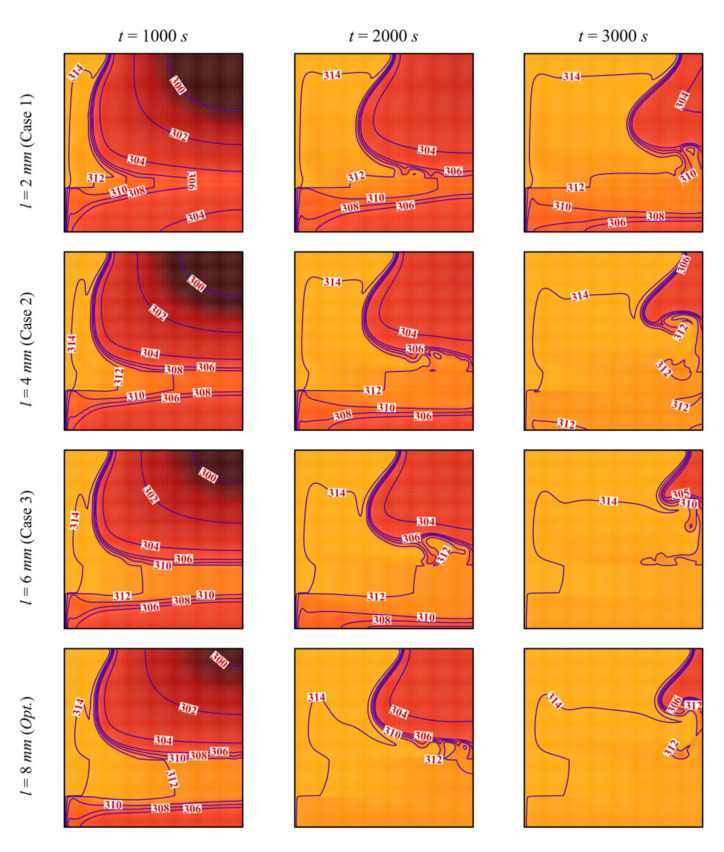
Isotherm lines for different control parameter at three times when *L* = 40 mm, *w* = 4 mm*_,_ h* = 11 mm, *ε* = 0.80, and *υ*_na_ = 0.04.

Figure 7 and Figure 8 depict the streamlines and the isotherms in the enclosure for different values of the porous layer width *l*, while keeping the other parameters at their optimal values. In all the cases, the PCM initially melts in the left-hand region of the cavity (near the heated wall). Two phenomena occur in the cavity: thermal convection of the melted PCM and enhancement of the heat transfer by the porous layer. Convective flow takes place where the heated fluid goes up and is replaced by a colder melt, leading to a clockwise circulation. The PCM melts around the porous layer in the initial stages of melting, and the extent of the region in which melting occurs increases with *l*. The high thermal conductivity of the porous layer enables the flow of heat through the PCM, and when the size of the layer is larger, more heat is transferred to the PCM, leading to enhanced melting. It can be seen that, at every instant, the zone of melted PCM is greater for a higher *l*. The isotherm contours show that the temperature is higher in the different zones of the cavity when *l* is increased, thus confirming the contribution of the size of the porous layer to the heat transfer. 

**Figure 9:**
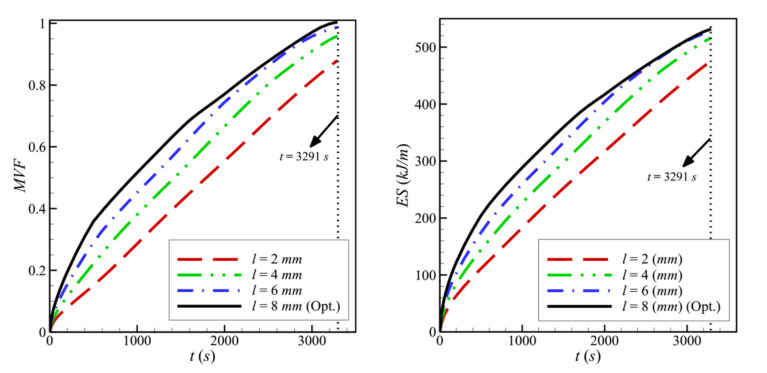
Variation of *MVF* (**left**) and *ES* (**right**) as a function of time for optimum porous layer thickness when *L* = 40 mm, *w* = 4 mm*_,_ h* = 11 mm, *ε* = 0.80, and *υ*_na_ = 0.04.

The variations of the *MVF* and the *ES* as a function of time are plotted in Figure 9 for various values of *l*. Full melting, corresponding to *MVF* = 1, is achieved more slowly when *l* is decreased. In addition, more energy is stored (*ES* increases) as *l* increases, leading to a higher charging power. This is due, as previously explained, to the enhanced heat transfer when the size of the porous layer is increased.

**Figure 10:**
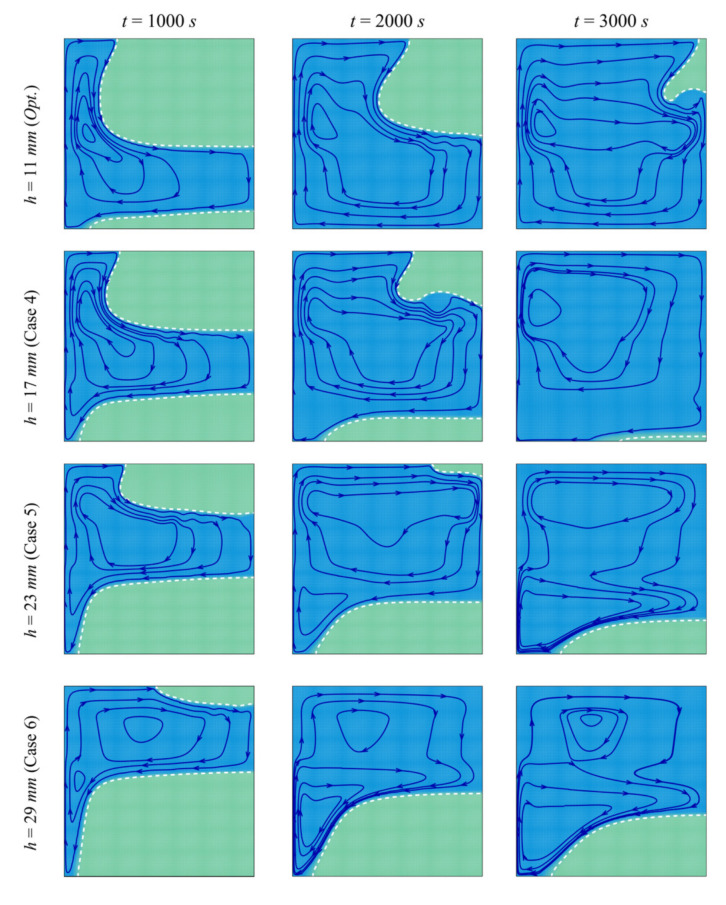
Streamlines and interface of melting (white dashed line) for different control parameter at three times when *L* = 40 mm, *w* = 4 mm, *l* = 8 mm, *ε* = 0.80, and *υ*_na_ = 0.04.

**Figure 11:**
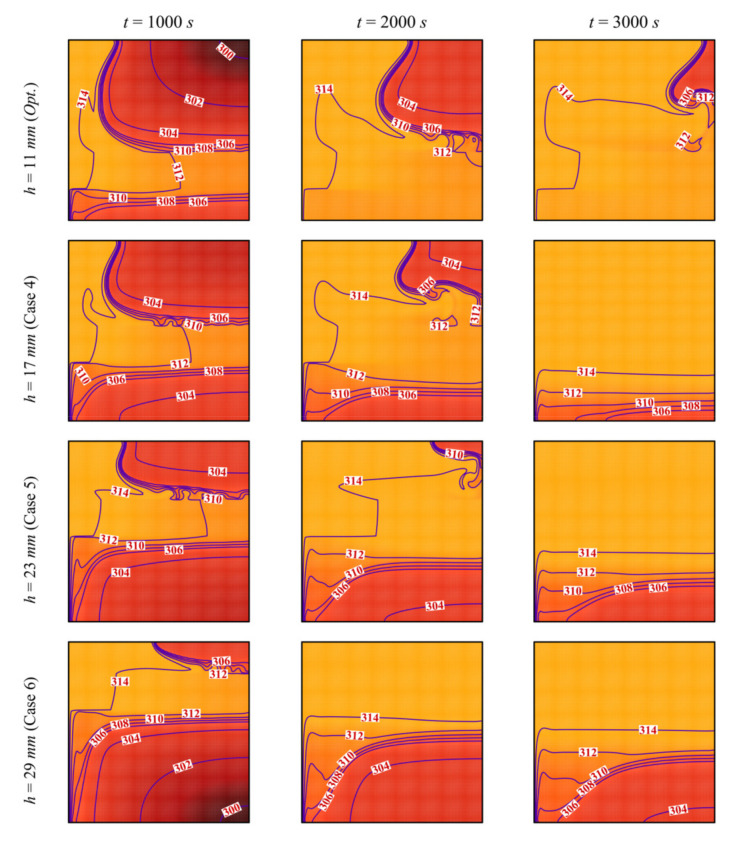
Isotherm lines for different control parameter at three times when *L* = 40 mm, *w* = 4 mm, *l* = 8 mm, *ε* = 0.80, and *υ*_na_ = 0.04.

The impact of the height, *h*, at which the porous layer is located above the bottom edge of the cavity on the streamlines and the isotherms is illustrated in Figure 10 and Figure 11. The same phenomena of thermal convection of the PCM and heat transfer enhancement by the porous layer are observed. However, it can be seen that moving the porous layer upwards (increasing *h*), even though still contributing to heat transfer, reduces PCM melting in the bottom part of the enclosure due to the diminished convective effects in that region. In fact, the heated PCM circulates to the top due to convection. When the porous layer is moved upwards, it increases the heat transfer to the (already hot) PCM, while moving it downwards contributes to heating the cold PCM and enhances thermal convection. This can be verified by looking at the isotherms, which clearly show that the bottom part becomes substantially colder when the porous layer is moved upwards.

**Figure 12:**
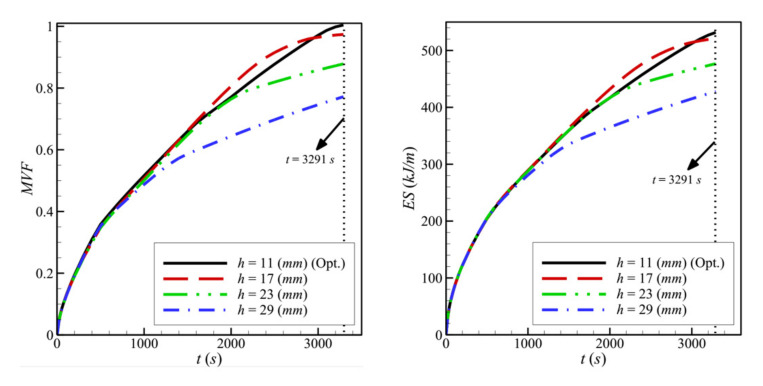
Variation of *MVF* (**left**) and *ES* (**right**) as a function of time for the optimum height porous layer when *L* = 40 mm, *w* = 4 mm, *l* = 8 mm, *ε* = 0.80, and *υ*_na_ = 0.04.

Figure 12 shows the variations of the *MVF* and the *ES* as a function of time for different values of *h*. Due to the hindered convection for higher values of *h*, the values of *MVF* and *ES* are both low when *h* is increased. Full melting occurs fast, and high charging power could be obtained when *h* is reduced.

**Figure 13:**
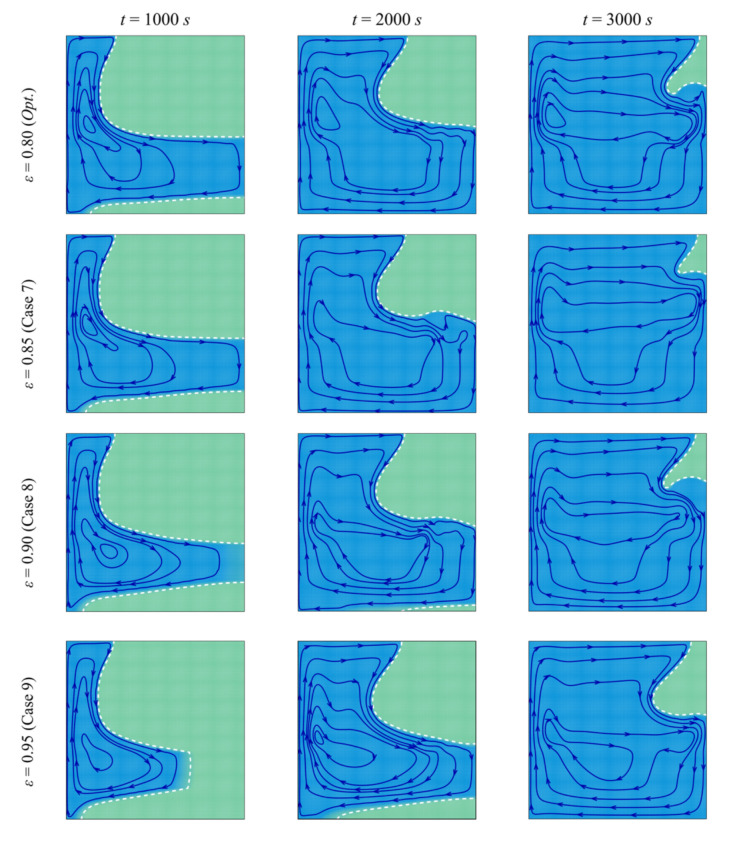
Streamlines and interface of melting (white dashed line) for different control parameter at three times when *L* = 40 mm, *w* = 4 mm*, l* = 8 mm, *h* = 11 mm, and *υ*_na_ = 0.04.

**Figure 14:**
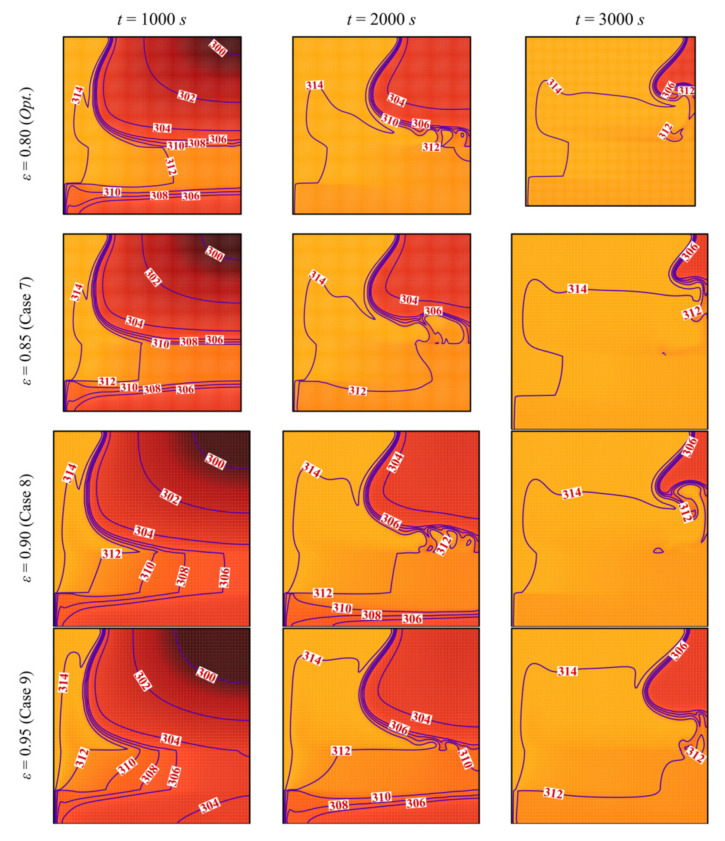
Isotherm lines for different control parameter at three times when *L* = 40 mm, *w* = 4 mm, *l* = 8 mm, *h* = 11 mm, and *υ*_na_ = 0.04.

**Figure 15:**
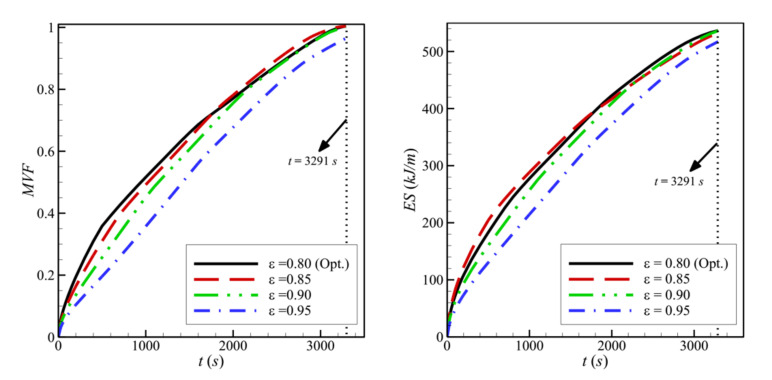
Variation of *MVF* (**left**) and *ES* (**right**) as a function of time for optimum porosity when *L* = 40 mm, *w* = 4 mm, *l* = 8 mm, *h* = 11 mm, and *υ*_na_ = 0.04.

The streamlines and the isothermal contours are depicted in Figure 13 and Figure 14 for various values of the porosity of the porous layer, *ε*. The area of melted PCM around the porous layer decreases when *ε* increases. For *ε* = 0.95, it can be seen that the zone of the melted PCM is considerably limited compared to the case of *ε* = 0.8. This indicates a weaker heat transfer when the porosity is increased. Indeed, the main factor that gives the porous layer the capacity to transfer heat is the high thermal conductivity of its solid matrix. *ε* represents the ratio of the void space in the porous layer compared to the total volume. When *ε* is increased, the size of the solid matrix is reduced, and the contribution of the porous layer to the heat transfer is diminished. The isothermal lines show that the temperature in each zone of the cavity is higher when *ε* is reduced, indicating an enhanced heat transfer. For these reasons, both the *MVF* and the *ES* decrease when *ε* is increased and are minimum for *ε*=0.95, as shown in Figure 15.

**Figure 16:**
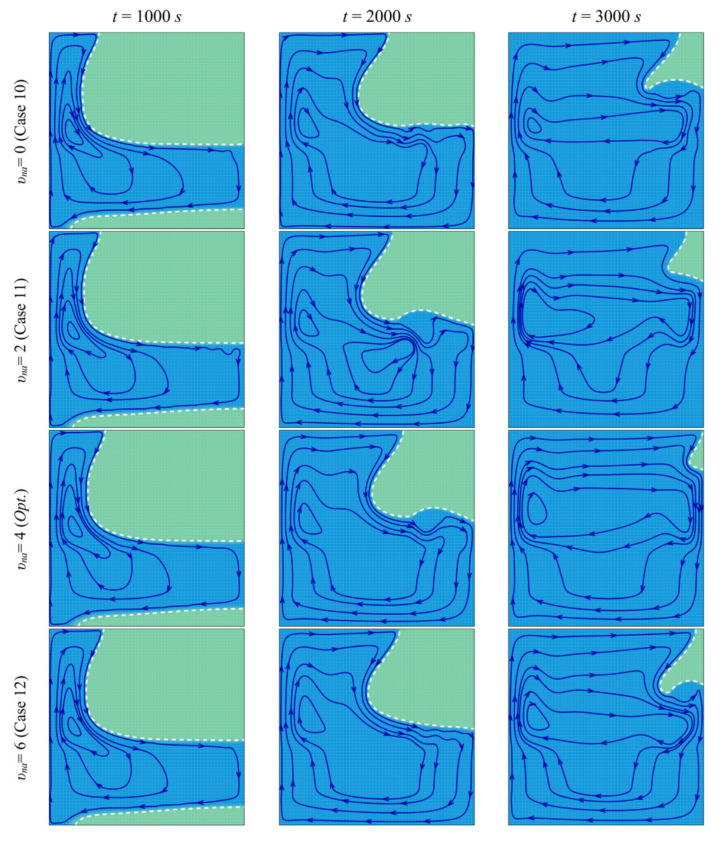
Streamlines and interface of melting (white dashed line) for different control parameter at three times when *L* = 40 mm, *l* = 8 mm, *h* = 11 mm, *ε* = 0.80, and *w* = 4 mm.

**Figure 17:**
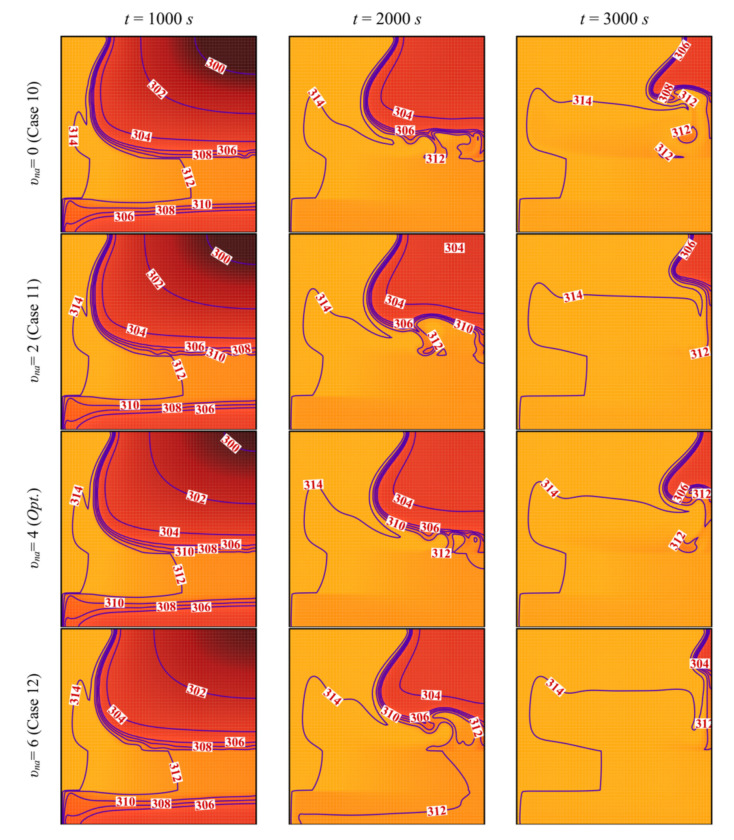
Isotherm lines for different control parameter at three times when *L* = 40 mm, *l* = 8 mm, *h* = 11 mm, *ε* = 0.80, and *w* = 4 mm.

**Figure 18:**
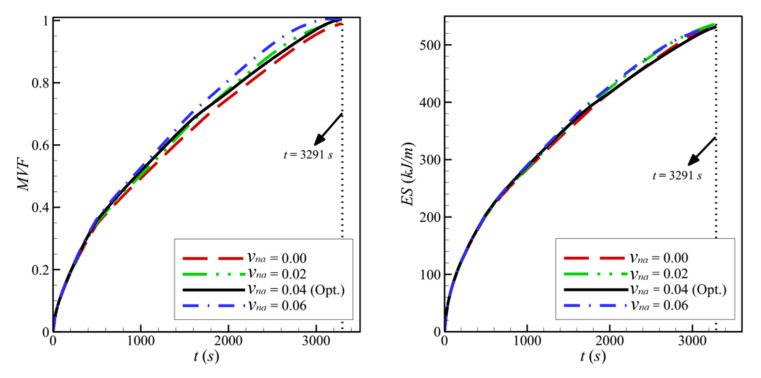
Variation of *MVF* (**left**) and *ES* (**right**) of copper nano-additives as a function of time for optimum wall thickness of Copper when *L* = 40 mm, *l* = 8 mm, *h* = 11 mm, *ε* = 0.80, and *w* = 4 mm.

Figure 16 and Figure 17 show a very limited effect of the volume fraction of nanoparticles, *v_na_*, on the flow patterns and the isothermal contours. In fact, as the porous layer’s location, size, and porosity are the same in all the cases, the geometry does not change when the volume fraction of the particles is varied. The variations of the MVF and the ES as a function of time are shown in Figure 18. It can be seen that the full melting is reached simultaneously for all the values of *v_na_*. Moreover, the values of *ES* show limited change with the variation of *v_na_*. In addition, compared to Figure 9, Figure 12, and Figure 15, Figure 18 shows that the impact of the nanoparticles’ concentration, *v_na_*, is limited on the values of *MVF* and *ES*, thus confirming the outcomes of Table 6, in which *v_na_* was shown to have a rank-four importance compared to the other control factors. 

## 5. Conclusions

The Taguchi method has been used to find the optimal combination of parameters to achieve the fastest melting of the PCM. The relative importance of each parameter was assessed. A total of 16 simulations were performed to enable the optimization. Using the optimized parameters as a base case, the parameters were varied to test if the Taguchi method had successfully found the optimum parameters and to understand how each control factor affects the thermal behavior of the PCM in the cavity. The main outcomes of the present investigation are summarized as follows:

- In a cavity with side length, *L* = 40 mm, the optimal values obtained for the control factors are the following: *w* = 4 mm, *l* = 8 mm, *h* = 11 mm, *ε* = 0.8, and *υ*_na_ = 0.04. By their decreasing order of influence, the control factors are ranked as: *h* > *ε* > *l* > *υ*_na_ > *w*. The variation of the design parameters could induce a 58% variation in the melting time.
- When the left wall is heated, PCM starts melting, and convective flow takes place. The presence of the porous layer in the cavity improves heat transfer and contributes to PCM melting.
- The size and location of the porous layer affect the thermal behavior of the PCM. Increasing the layer size, *l*, enhances and accelerates heat transfer. The charging power increases with *l*. Just shifting the porous layer from 2 mm to 6 mm increased the melting rate by 8.8%.
- Moving the porous layer upwards (increasing *h*) hinders the convective effects in the bottom part of the cavity and lowers the contribution of the porous layer to PCM melting. The charging power decreases when *h* is raised.
- Reducing the porosity of the porous layer, *ε*, which is equivalent to a higher presence of the solid matrix and, consequently, a higher thermal conductivity, enhances heat transfer and PCM melting. The charging power increases as *ε* decreases.
- The width of the heated copper wall, *w*, has a very limited effect on the charging power.

In the present study, the foam layer could be placed horizontally, and its location could change in a horizontal direction. However, it is also possible to place the foam layer vertically and change its location horizontally. The layer can also be placed along the enclosure walls. These designs can be subject to future investigations. 

## Figures and Tables

**Table 1:** Thermophysical values of the PCM and the copper nanoparticles [41,42].

Properties	Capric Acid	Copper
Density (kg m^−3^)	Solid: 1018 Liquid: 888	8933
Kinematic viscosity (m^2^ s^−1^)	3 × 10^−6^	N/A
Thermal expansion coefficient (K^−1^)	1 × 10^−3^	1.67 × 10^−5^
Thermal conductivity (Wm^−1^ K^−1^)	Solid: 0.372 Liquid: 0.153	401
Latent heat (kJ kg^−1^)	152.7	N/A
Phase change temperature (°C)	32	N/A
Specific heat (kJ kg^−1^ K^−1^)	Solid: 1.9 Liquid: 2.4	0.385

N/A: Not applicable.

**Table 2:** Details of uniform grid check cases in which specified conditions are *L* = 40 mm, *w* = 3 mm, *l* = 6 mm, *h* = 11 mm, *ε* = 0.85, and *υ*_na_ = 0.06.

Cases	Mesh Size in Wall	Mesh Size in PCM	$\mathrm{MVF}\left|{}_{t=2000s}\right.$	Computational Time
Case I	4 × 75	75 × 75	0.7281	14 h 19 min 15 s
Case II	5 × 100	100 × 100	0.7273	9 h 45 min 20 s
* Case III	6 × 125	125 × 125	0.7327	10 h 13 min 36 s
Case IV	7 × 150	150 × 150	0.7369	10 h 14 min 17 s
Case V	8 × 175	175 × 175	0.7401	12 h 33 min 11 s

* the case was selected for computations.

**Table 3:** The range and levels of the control parameters.

Factors	Description	Level 1	Level 2	Level 3	Level 4
**A**	*w* / mm (Copper wall thickness)	1	2	3	4
**B**	*l* / mm (Porous layer thickness)	2	4	6	8
**C**	*h* / mm (Porous layer height)	11	17	23	29
**D**	*ε* (Porosity)	0.80	0.85	0.90	0.95
**E**	*υ*_na_ (Nanoparticles volume fraction)	0.00	0.02	0.04	0.06

**Table 4:** Taguchi orthogonal table corresponding to the range and levels of the control parameters.

Case No.	Control Parameters					*MVF* = 1		
	A	B	C	D	E	t|*_MVF_*_=1_ / s	* CP / J m^−1^ s^−1^	S/N Ratio
	*w* / mm	*l* / mm	*h* / mm	*ε*	*υ_na_*			
1	1	2	11	0.8	0	4100	131.5572	−72.2557
2	1	4	17	0.85	0.02	4700	113.2655	−73.4420
3	1	6	23	0.90	0.04	5800	91.0539	−75.2686
4	1	8	29	0.95	0.06	6700	78.6821	−76.5215
5	2	2	17	0.90	0.06	5600	93.6736	−74.9638
6	2	4	11	0.95	0.04	4400	122.7105	−72.8691
7	2	6	29	0.8	0.02	6300	80.5411	−75.9868
8	2	8	23	0.85	0	5600	95.4117	−74.9638
9	3	2	23	0.95	0.02	7900	68.6977	−77.9525
10	3	4	29	0.90	0	7800	68.6409	−77.8419
11	3	6	11	0.85	0.06	3300	159.8793	−70.3703
12	3	8	17	0.8	0.04	3400	153.9024	−70.6296
13	4	2	29	0.85	0.04	6900	73.5752	−76.7770
14	4	4	23	0.8	0.06	5100	98.5855	−74.1514
15	4	6	17	0.95	0	5600	98.7377	−74.9638
16	4	8	11	0.90	0.02	3400	159.6877	−70.6296

* CP is the power of the stored energy (stored energy/Time).

**Table 5:** The optimum values of the controlling parameters.

Optimum Factors					Optimum Melting Time at *MVF* = 1	
*W*	*L*	*H*	*ε*	*υ*_na_	Taguchi Prediction	Tested Case
4 mm	8 mm	11 mm	0.80	0.04	2025s	3291.6

**Table 6:** Deviations and ranks of the controlling parameters.

	*w* / mm	*l* / mm	*h* / mm	*ε*	*υ*_na_
Level 1	−74.37	−75.49	−71.53	−73.26	−75.01
Level 2	−74.70	−74.58	−73.50	−73.89	−74.50
Level 3	−74.20	−74.15	−75.58	−74.68	−73.89
Level 4	−74.13	−73.19	−76.78	−75.58	−74.00
*δ*	0.57	2.30	5.25	2.32	1.12
Rank	5	3	1	2	4

**Table 7:** Further analysis around the optimum case (*w* = 4 mm, *l* = 8 mm, *h* = 11 mm, *ε* = 0.8, and *υ*_na_ = 0.04).

Case No.	Parameter	Control Parameters					at t = 3291.6 s	
		A	B	C	D	E	MVF	^*^CP / J m^−1^ s^−1^
		*w* / mm	*l* / mm	*h* / mm	*ε*	*υ*_na_		
1	*l*	4	2	11	0.80	0.04	0.9171	144.6119
2		4	4	11	0.80	0.04	0.9845	156.9111
3		4	6	11	0.80	0.04	0.9976	160.5965
4	*h*	4	8	17	0.80	0.04	0.9998	158.3983
5		4	8	23	0.80	0.04	0.9317	144.8368
6		4	8	29	0.80	0.04	0.8469	130.0390
7	*ε*	4	8	11	0.85	0.04	1.0000	163.0727
8		4	8	11	0.9	0.04	0.9982	162.9335
9		4	8	11	0.95	0.04	0.9635	157.2007
10	*υ*_na_	4	8	11	0.80	0.0	0.9856	163.2366
11		4	8	11	0.80	0.02	0.9961	163.2134
12		4	8	11	0.80	0.06	1.0000	160.8724

## Data Availability

The data will be available on request.
